# Value of transrectal ultrasound-guided biopsy in endoscopy negative biopsy patients with rectal lesions

**DOI:** 10.1186/s12876-023-02806-3

**Published:** 2023-05-22

**Authors:** Tingting Li, Man Lu, Yuan Li, Wei Yang

**Affiliations:** Department of Ultrasound, Sichuan Cancer Hospital & Institute, Affiliate Cancer Hospital of University of Electronic Science and Technology of China (UESTC), Chengdu, China

**Keywords:** Endoscopy biopsy, Colorectal cancer, Transrectal ultrasound-guided biopsy, CEUS

## Abstract

**Objective:**

To evaluate the value of transrectal ultrasound (TRUS) guided biopsy in endoscopy negative biopsy patients with rectal lesions.

**Methods:**

150 endoscopy negative biopsy result rectal lesions adopted the transrectal ultrasound-guided biopsy. Based on whether the patients received contrast-enhanced ultrasound examination or not before the biopsies, all enrolled cases were divided into TRUS guided group and contrast-enhanced TRUS (CE-TRUS) guided group, and the safety and diagnostic performances were analyzed retrospectively.

**Results:**

We obtained adequate specimens in the majority of cases (98.7%,148/150); There were no complications identified in our study. 126 patients received contrast-enhanced TRUS examination before biopsy to evaluate vascular perfusion and necrosis. The sensitivity, specificity, PPV, NPV, and overall accuracy of all biopsies were 89.1%, 100%,100%, 70.4%, and 91.3% respectively; The sensitivity, specificity, PPV, NPV, and accuracy for TRUS-guided biopsy and CE-TRUS guided biopsy were 73.7%, 100%,100%, 50%,79.2% and 92.1%,100%,100%,75%,93.6% separately; The increase in correct diagnoses was significant (p < 0.05) between TRUS guided biopsy and CE-TRUS guided biopsy.

**Conclusion:**

TRUS-guided biopsy is a reliable procedure that can be augmented by endoscopic biopsy techniques if the biopsy yields negative results. CE-TRUS might assist in the location of the biopsy and decrease sampling errors.

## Introduction

Colorectal cancer was fifth cancer in both incidence and mortality in China [1, 2]. In both China and the USA, the overall population’s colorectal cancer rates and burden have increased [3, 4]. The available treatments for rectal cancer are determined by an accurate histological diagnosis. Before performing resectional surgery for early-stage rectal cancer, surgeons have traditionally performed a biopsy; nevertheless, in cases of advanced tumors requiring neoadjuvant chemotherapy or radiation therapy before surgery, the biopsy is necessary to confirm the presence of cancer. Local recurrence is still a serious issue in individuals who have had curative resection of rectal cancer, occurring in 15–25% of cases. Early pathologic detection of recurrence is required for subsequent decision-making and treatment options [5, 6].

Today, endoscopic biopsy, with its excellent specificity of close to 100%, is the primary technique utilized to collect a tissue sample for diagnosis. But according to the research that reported endoscopic biopsy sensitivities, the sensitivities range significantly from 50 to 100% depending on different procedures, the number of samples taken, and the various tissue volumes obtained. [7–9]. If the affected area is not sampled, the specimens might not be indicative of the final histology, and a negative biopsy may not help rule out invasive disease in some large, apparently benign lesions [10]. In this patient, the repeated tissue sample is frequently necessary [11–13]. Repeat endoscopy entails anesthetic and procedural hazards. In about 80% of patients, pelvic or perianastomotic recurrences of the rectal tumor coexist with local recurrences, these sites are not always accessible for endoscopy biopsy [5]. Herein, a new biopsy method might urgently need in this endoscopy negative biopsy patient.

Rectal lesions are frequently diagnosed with transrectal ultrasound (TRUS). TRUS may clearly show the five layers of the intestinal wall, allowing for assessment of the depth of infiltration of lesions and evaluation of pathological conditions. TRUS-guided core biopsy, however, is a rather uncommon operation in rectal cancers. Since its first introduction and application in the prostate in 1989, TRUS-guided core biopsy has rapidly expanded and is now the gold standard for the early diagnosis of prostate cancer [14]. The effectiveness of TRUS-guided biopsy has been confirmed mostly in local rectal cancer recurrence in research related to TRUS-guided biopsy in rectal cancer [15–17]. The target lesion’s blood flow and vascular perfusion are visible on contrast-enhanced ultrasonography (CEUS). The use of CEUS in TRUS-guided prostate biopsy reduces the number of biopsy cores required for diagnosis confirmation by enhancing the detection and localization of malignant tumors by ultrasound [18–20]. Therefore, the purpose of this study is to evaluate the prospective value of TRUS-guided core biopsy in endoscopy negative biopsy patients with the help of CEUS or not.

## Materials and methods

### Patients

This study was approved by the Institutional Review Board and Ethics Committee of Sichuan Cancer Hospital (SCCHEC-03-2018-029). All patients signed written informed consent before the examination.

From June 2018 to March 2022, a total of 150 consecutive patients were retrieved in this study. The inclusion criteria were as follows: (1) all rectal lesions had endoscopy negative biopsy pathology results before TRUS guided biopsy; (2) the distal border had to be＜15 cm above the anal verge; (3) patients had to have normal preoperative regular laboratory examinations, and (4) true positive biopsy results had to be histologically confirmed postoperatively; The exclusion criteria were as follows: patients with incomplete clinical and pathologic characteristics.

### TRUS and Contrast-enhanced TRUS examination (CE-TRUS)

According to the patient’s condition and willingness to take a contrast-enhanced TRUS before the biopsy process, all patients were divided into the TRUS group and the CE-TRUS group. All patients underwent a TRUS (EPIQ 7 and EPIQ 5 ultrasound system, Philips Healthcare, Bothell, WA, USA, with a C10-3 V endocavitary transducer) by an experienced radiologist (Man Lu, with more than 10 years of experience in gastrointestinal US, CEUS, and intervention). In patients without contraindications for contrast agent and willing to take CE-TRUS, when the suspicious lesions were found, a 2.4ml bolus of Sonovue (BraccoSpA, Milan, Italy; made up of sulfur hexafluoride (SF6)-filled microbubbles) was injected intravenously, followed by a 5-mL saline flush. The radiologist observed the enhancement of the lesion and identified the enhanced area and unenhanced area. The unenhanced area in the lesion showed the cystic part of the lesion and should be avoided during the biopsy procedure.

### TRUS-guided biopsy and CE-TRUS guided biopsy

When the biopsy target area was determined on CE-TRUS, the radiologist prepared the disinfection as well as the drape. After local anesthesia with 2% lidocaine, using a needle guide device attached to the transducer shaft, an 18-gauge automatic core biopsy needle (Magnum and Max-Core, Bard, Tempe, AZ, USA) was advanced to the lesion and performed the biopsy. The penetration depth was set to 15- or 22 mm depending on the size and location of the lesion. We aimed to take two to four samples in all patients. All the sampled specimens were fixed in 8% formaldehyde solution and labeled immediately and were analyzed by routine histologic staining and immunohistochemistry if required.

### Statistical analysis

Data were analyzed using SPSS 22.0 (SPSS Inc., Chicago, IL, USA). Categorical data were presented with frequency and percentage. Continuous data were presented with mean and standard deviation. By testing against the pathological result, the diagnostic sensitivity, specificity, positive predictive value (PPV), negative predictive value (NPV), and accuracies of CE-TRUS guided biopsy and TRUS guided biopsy were calculated. P < 0.05 was considered statistically significant in all analyses.

## Results

A total of 150 cases were enrolled in this study ranging in age from 27 to 84 years (mean ± SD, 57.2 ± 11.0 years). The median lesion size was 4.5 cm (range, 0.9 to 13.0 cm). We obtained adequate specimens in the majority of cases (98.7%,148/150); in 2 (1.3%) cases the specimen was not suitable for histological diagnosis due to insufficient material. The most common biopsy site was the lower rectum (67.3%, 101/150). 20% (30/150) cases were submucosal lesions with intact mucosa protruding into the cavity (Table 1). 80% (120/150) cases showed visible mucosal damage, and 3–5 biopsies were taken each time in the endoscopy process.

**Table 1:** Patient and lesion characteristics

Variables	Description (N = 150)
Sex (Male/Female)	102/48
Age	57.2 ± 11.0
Size(malignant/benign)	2.9 ± 2.3 cm/2.1 ± 1.2 cm
Height in the rectum (Lower/Medium/Upper)	101/15/34
Malignant (Adenocarcinoma/ GIST/SCC/NET/ sarcoma/ mesothelioma)	95/10/7/6/1/1
Benign (Adenoma/ Inflammatory / hemangioma/endometriosis)	7/21/1/1

The majority of biopsy cases (79.3%, 119 /150) were malignancy in this study, and these cases were constituted by suspicion of primary malignancy in 110 cases (91.6%, 109/119); suspicion of rectal recurrence in 5 cases (4.2%, 5/119); suspicion of metastasis in 5 cases (4.2%, 5/119), 1 metastatic carcinoma of the ovary, 1 cervical cancer metastasis and 3 prostate cancer metastasizes.

A total of 126 patients received contrast-enhanced TRUS examination before biopsy to evaluate the vascular perfusion and necrosis area. Taking the neighboring normal rectal wall as a reference, the contrast-enhanced conditions of the lesion were classified as hypo-enhanced, hyper-enhanced, iso-enhanced, and non-enhanced. Hyper-enhanced presented mostly in all cases (84.9%, 107/126), hypo-enhanced(11.1%, 14/126), iso-enhanced (1.6%, 2/126), and non-enhanced (2.4%, 3/126).

**Fig. 1:**
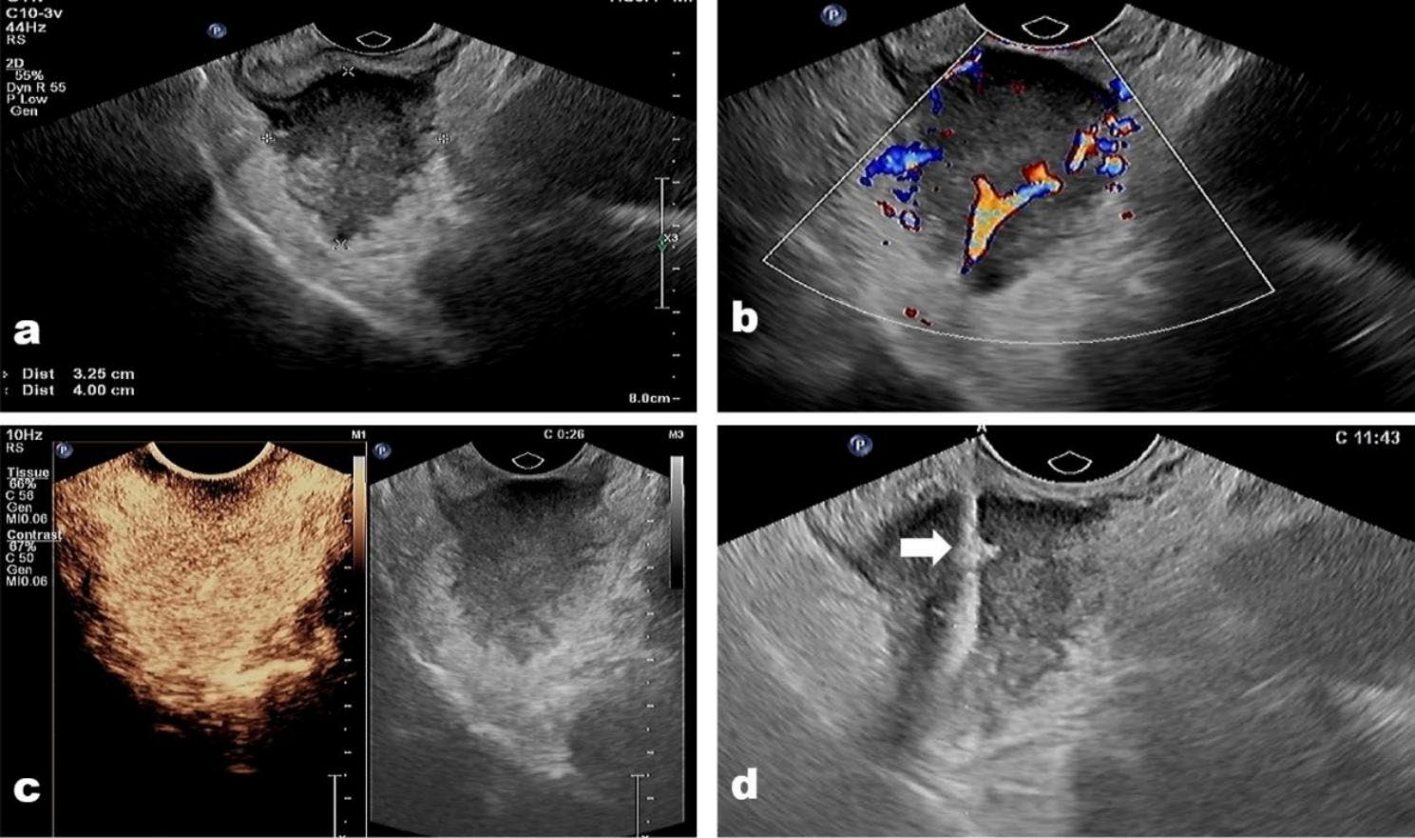
**(a)** A neuroendocrine neoplasm located in the lower rectum in a 51-year-old man. **(b)** The tumor showed abundant vascular on color Doppler image; **(c)** the tumor presented hyper-enhanced on contrast-enhanced condition, and there was no necrosis area in the tumor. **(d)** The biopsy needle (arrow) passes through the tumor center to acquire biopsy samples

**Fig. 2:**
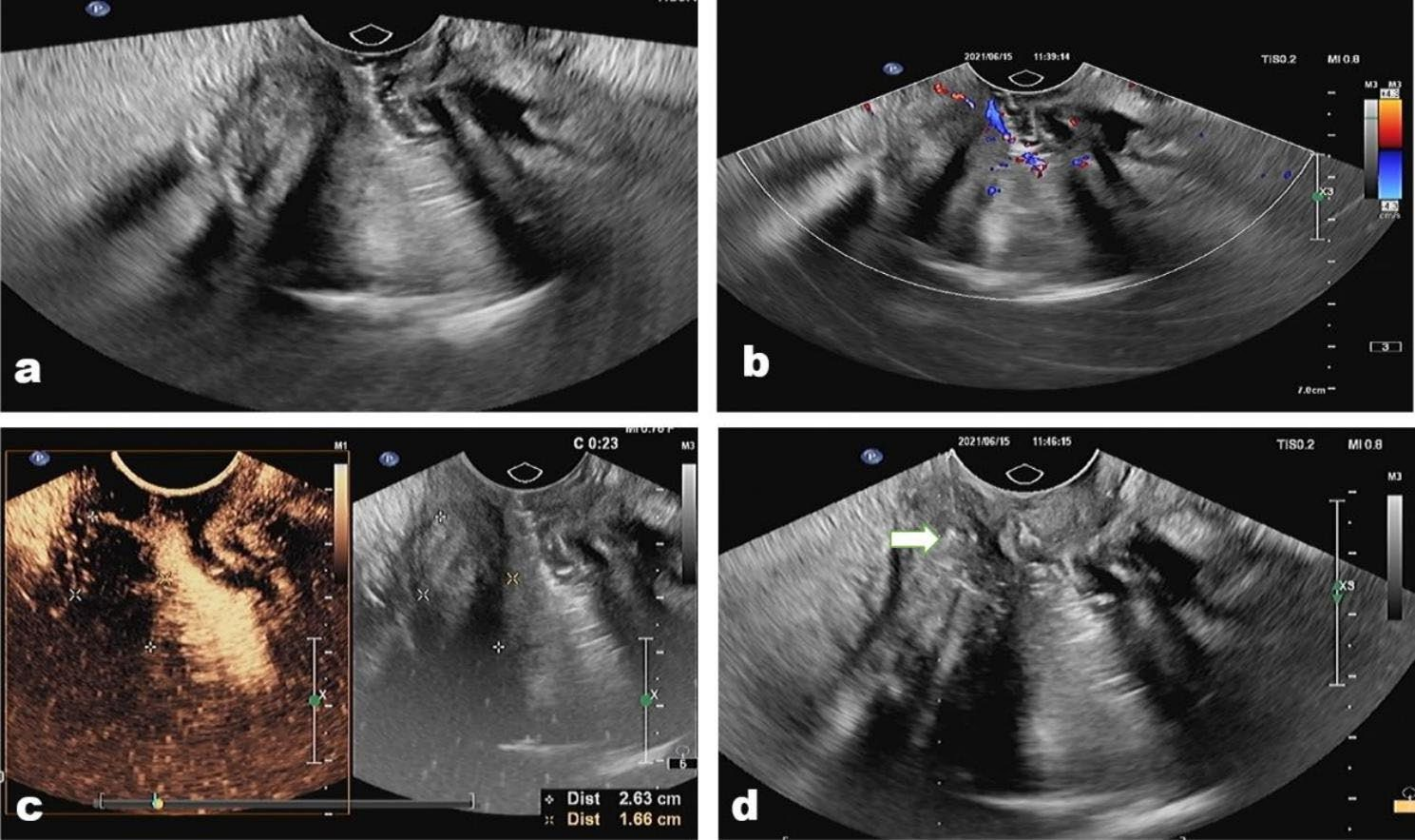
**(a)** An inflammatory lesion located in the medium rectum in a 46-year-old man. **(b)** The lesion showed an absence of vascularity in the lesion but abundant vascularity in the peripheral tissue on color Doppler image; **(c)** the lesion presented non-enhanced on contrast-enhanced condition; **(d)** The biopsy needle (arrow) pass from the basal of the lesion to acquire biopsy samples both from the lesion and peripheral tissue

In this study, histological inaccuracies were found in 13 cases (8.7%). The sensitivity, specificity, PPV, NPV, and overall accuracy of all biopsies were 89.1%, 100%, 100%, 70.4%, and 91.3% respectively (Table 2). The sensitivity, specificity, PPV, NPV, and overall accuracy for TRUS-guided biopsy and CE-TRUS guided biopsy were 73.7%, 100%, 100%, 50%, 79.2%, and 92.1%, 100%, 100%, 75%, 93.6% separately; The increase in correct diagnoses was significant (p < 0.05) between TRUS guided biopsy and CE-TRUS guided biopsy (Table 2) (Figs. 1 and 2). The pathology characteristics of misdiagnosed patients were listed in Table 3.

**Table 2:** The sensitivity, specificity, PPV, NPV, and accuracy of overall biopsy, TRUS-guided biopsy, and CE-TRUS guided biopsy

	Sen(%)	Spe(%)	PPV(%)	NPV(%)	Accuracy(%)
Overall biopsy	89.1	100	100	70.4	91.3
95%(CI)	(81.7,93.8)	(86.2,100)	(95.6,100)	(54.6,82.7)	— —
TRUS guided	73.7	100	100	50	79.2
95%(CI)	(52.3,92.1)	(48.6,89.9)	(73.2,100)	(20.1,79.8)	— —
CE-TRUS guided	92.1	100	100	75	93.6*
95%(CI)	(84.7,96.3)	(82.8,100)	(95.1,100)	(56.2,87.9)	— —

CI: confidence interval; Sen: sensitive; Spe: specificity; NPV: negative predictive value; PPV: positive predictive value;

*The increase in correct diagnoses was significant (p < 0.05) between TRUS-guided biopsy and CE-TRUS guided biopsy

**Table 3:** The pathology characteristics of misdiagnosed patients

Histology of Biopsy	Histology of Surgery	amount	CT/MRI results
villous adenoma	Rectal adenocarcinoma	4	Tumor lesion
Inflammatory	Rectal adenocarcinoma	5	Inflammatory tissue/ Rectal adenocarcinoma
fibrous connective tissue	Rectal adenocarcinoma	2	Thickened intestinal wall
Inflammatory	Prostate cancer	1	Prostate cancer
Inflammatory	Squamous carcinoma	1	Tumor lesion

## Discussion

Endoscopy plays an important role in a rectal tumor tissue biopsy, while negative biopsy results happened in some cases occasionally [9]. Large tumors, some submucosal tumors, and tumor liquefaction could all affect the endoscopic biopsy results. In this study, we examined the diagnostic efficacy and safety of TRUS-guided biopsy and CE-TRUS-guided biopsy for rectal lesions in 150 cases with negative biopsy results or inability to get tissues via endoscopy. We obtained adequate specimens for histopathology diagnosis in more than 95% of cases, while there were no major complications either during the biopsy process or post-biopsy.

This study is, to date, the largest reported series of ultrasound-guided biopsies in rectal lesions with endoscopy negative biopsy results. In every patient, the diagnostic accuracy was higher than 90%. Additionally, the specificity of 100% for both TRUS-guided biopsy and CE-TRUS guided biopsy patients demonstrated that these biopsy procedures appeared to be extremely specific for diagnosing malignancy. These results were also highly consistent with endoscopic biopsy results. The sensitivity for ultrasound-guided biopsy reached 89.1% in this study. According to earlier research, the sensitivity of an endoscope biopsy ranged from 50 to 100%, and it was strongly connected with the total number of biopsies. According to earlier research, the sensitivity ranged between 50% and 86% when 3 or 4 biopsies were taken and climbed to 100% when up to 10 biopsies were taken. In contrast to earlier flexible or stiff endoscopic biopsies, our study found that a median of 3 samples can achieve comparatively higher sensitivity, however, these biopsies are frequently too superficial to detect invasive growth [8, 21].

Additionally, the sensitivity and accuracy of the CE-TRUS guided biopsy were greater than those of the TRUS-guided biopsy (92.1% versus 73.7% for sensitivity and 93.6% versus 79.2% for accuracy). The lesion’s distribution of vascularity was shown by CEUS; the non-enhanced area in the CEUS image represented the necrotic region of the lesion and should be avoided during the biopsy. The hyper-enhanced region should be sampled during the biopsy procedure since it typically indicates a plentiful blood supply. In our investigation, there was no statistically significant difference between the CE-TRUS group and the TRUS group in terms of lesion size, rectum height, or the number of biopsies. As a result, CEUS facilitates biopsy placement and reduces sampling errors.

In addition, we compared the histological results of the biopsies with subsequent surgical histological results, a total of 13 lesions’ biopsy results conflicted with the final surgery histology results. Regarding the misdiagnosed cases in this study, the misdiagnosed rate for CE-TRUS guided biopsy (6.3% (8/126)) is lower than TRUS guided biopsy (20.8% (5/24)). If the lesion is severely ulcerated or necrotic, sampling may not provide viable tumor tissue, and biopsy specimens may not produce a diagnosis that is consistent with the surgical histological findings [9]. The necrotic region of the tumor appeared non-enhanced on CE-TRUS, and this region needs to be avoided while planning the biopsy approach.

**Fig. 3:**
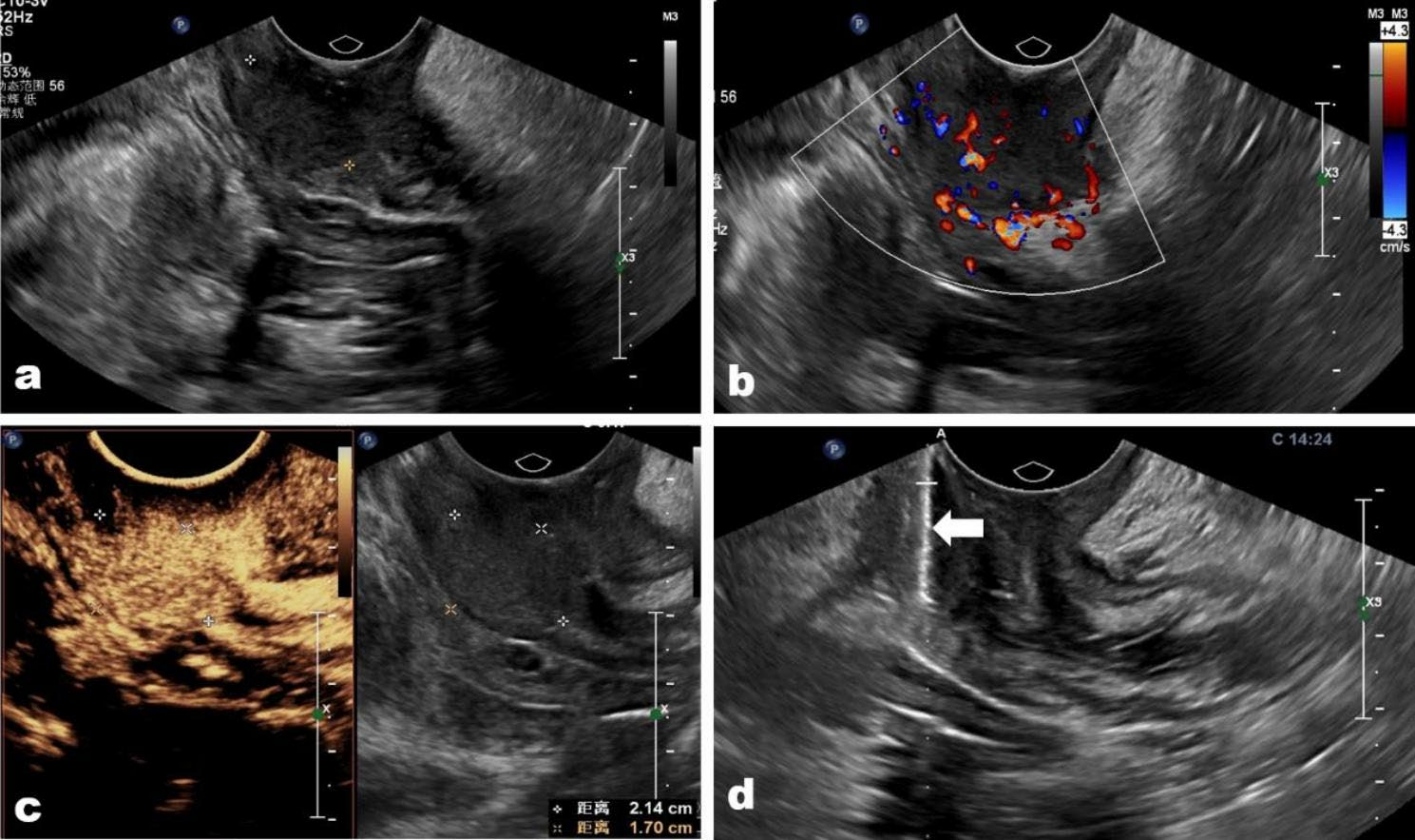
**(a)** A cervix metastasis squamous carcinoma located in the anal tube in a 43-year-old woman was misdiagnosed. **(b)** The tumor showed abundant vascular on color Doppler image; **(c)** the tumor presented hyper-enhanced on contrast-enhanced condition, and there was no necrosis area in the tumor. **(d)** The biopsy needle (arrow) passes through the tumor center to acquire biopsy samples

**Fig. 4:**
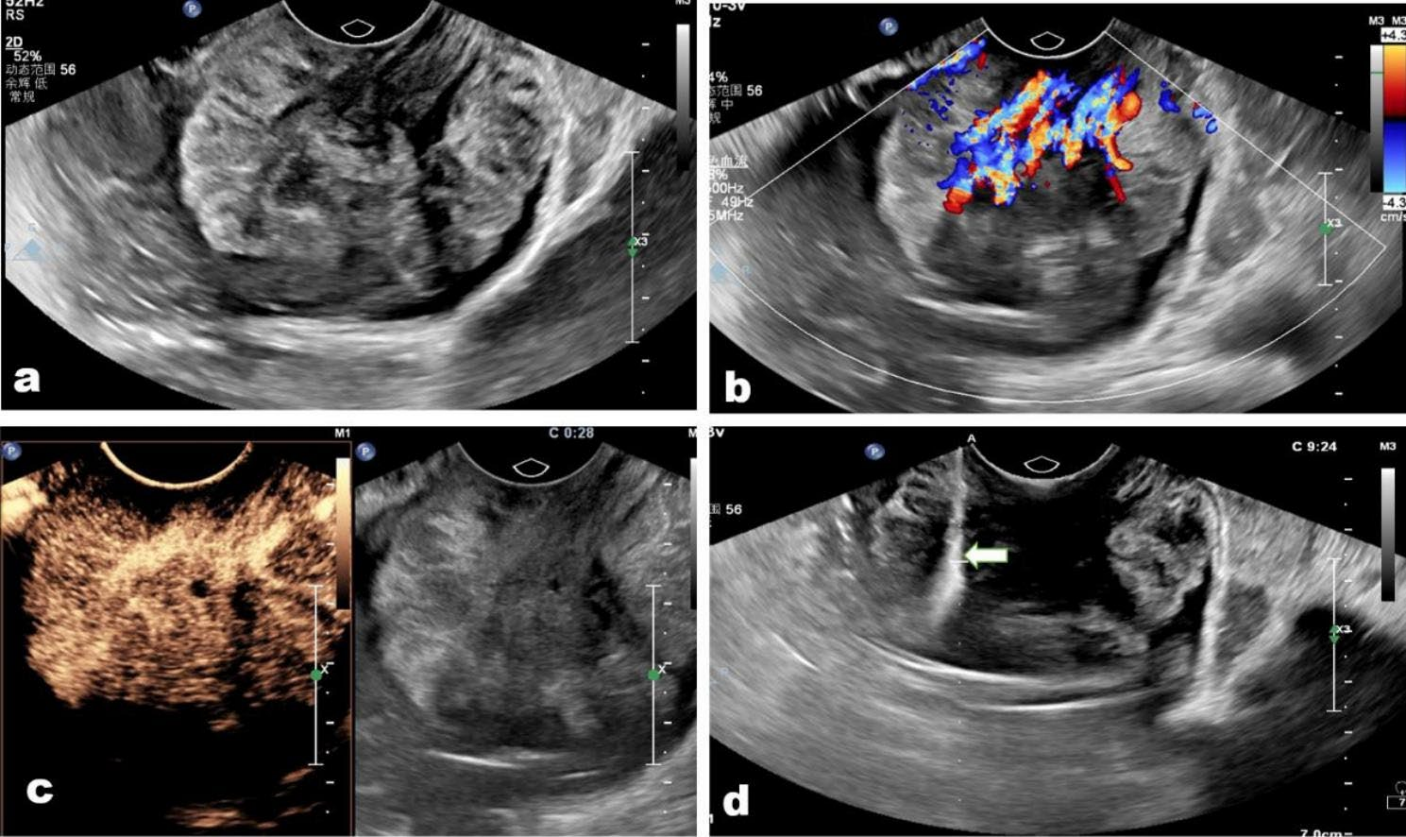
**(a)** A lower rectum rectal adenocarcinoma misdiagnosed as villous adenoma in a 48-year-old man. **(b)** The tumor showed abundant arborization vascular on color Doppler image; **(c)** the tumor presented regular hyper-enhanced on contrast-enhanced condition, and there were some minor necrosis areas in the tumor. **(d)** The biopsy needle (arrow) passes through the tumor center to acquire biopsy samples

According to the histologic diagnosis in cases of misdiagnosed CE-TRUS guided biopsy, three inflammatory disorders were recorded; nevertheless, the final diagnoses were adenocarcinoma, prostate cancer, and squamous carcinoma, each independently (Fig. 3). This discrepancy could be explained by the possibility that inflammatory conditions brought on by tumor cells spread muddle biopsy pathologic confirmation. The four additional rectal adenocarcinomas that resembled big polyps and were misdiagnosed as villous adenomas (Fig. 4) all had tumors that were larger than the average size in this study. We do believe that choosing a deeper biopsy location and acquiring more biopsy samples, similar to how endoscopy was repeated in 30% of patients with negative biopsies to obtain additional biopsy samples, could enhance the diagnosis accuracy in some big polyps [9].

Our findings show that either the CE-TRUS guided or TRUS-guided biopsy is technically simple and generally safe, given the paucity of literature on this subject. There were no complications identified in our study. By assessing the patient’s clinical indicators from routine blood assessments and post-biopsy surveillance, the safety of procedures was underlined; proper preparations are essential to achieve a low complication rate. Transrectal biopsy has a well-established history of using antibiotic prophylaxis to avoid infection. There were no complications identified in our study. The safety of procedures was emphasized by evaluating the patient’s clinical indications from routine blood assessment and post-biopsy observation, the adequate preparations are critical to ensure a low complication rate. Antibiotic prophylaxis is well-established in transrectal biopsy for the prevention of infection. Finally, whether CE-TRUS guided or TRUS-guided biopsies should be performed by experienced radiologists. The admission of this technique should be strictly regulated, in our department, where the radiologist who did the CE-TRUS guided or TRUS guided biopsies should have at least more than 8 years of expertise in gastrointestinal US, CEUS, and intervention.

We do think, however, the present study has several limitations. Firstly, this is a retrospective analysis based on our single-institution clinical experience, the valid and generalizable diagnostic performances of this technique require more results from different institutions. Additionally, because there were fewer biopsies in our study than endoscopic biopsies, it was possible to further assess the link between the number of samples and accuracy.

In conclusion, CE-TRUS guided or TRUS-guided biopsy is a dependable process that can be augmented by endoscopic biopsy techniques if the biopsy yields negative results. CE-TRUS might assist in the location of the biopsy and decrease sampling errors.

## Data Availability

All data generated or analyzed during this study are included in this published article.
